# A systematic review on the accuracy of zirconia crowns and fixed dental prostheses

**DOI:** 10.1080/26415275.2019.1708202

**Published:** 2020-01-07

**Authors:** Per Svanborg

**Affiliations:** Department of Prosthodontics/Dental Materials Science, Institute of Odontology, The Sahlgrenska Academy, University of Gothenburg, Göteborg, Sweden

**Keywords:** Zirconia, fixed dental prosthesis, accuracy

## Abstract

**Purpose:**

The aim of this study was to review the fit and assess the accuracy of tooth-supported single and multi-unit zirconia fixed dental prostheses.

**Background:**

The fit of zirconia restorations has been reported in several studies, but the accuracy of the manufacturing process is seldom discussed or used when drawing conclusions on the fit.

**Materials and methods:**

A literature search of articles published in PubMed between 2 March 2013 and 1 February 2018 was performed using clearly defined inclusion and exclusion criteria. 841 articles were found and 767 were excluded after screening the title and abstract. After full-text analysis another 60 articles were excluded which left 14 articles to be included for data extraction. Fit was the mean of distances reported in the studies and accuracy was the fit minus the pre-set spacer

**Results:**

For marginal gap of single crowns and multi-unit FDPs combined, the fit was 83 μm and the accuracy was 59 μm. The internal gap fit was 111 μm and the accuracy 61 μm. For the total gap, the fit was 101 μm, and the accuracy of the zirconia restorations was 53 μm.

**Conclusions:**

Within the limitations of the present systematic review the fit of zirconia single crowns and multi-unit FDPs may be regarded as clinically acceptable, and the accuracy of the manufacturing of zirconia is ∼60 μm for marginal, internal, and total gap. Also, digital impressions seem to be associated with a smaller gap value.

## Introduction

The fit of dental restorations is an important factor for the longevity of tooth-supported dental prostheses. A poor fit can affect the cement junction and result in dissolution, which may result in the loosening of the restoration or secondary caries [1]. Also, crowns with the poor marginal fit on subgingivally placed margins may increase bacterial retention and cause gingival inflammation [2].

There is no consensus on what is regarded as clinically acceptable fit, for marginal fit several authors suggest ∼100 μm [1,3–6]. For internal fit, McLean and von Fraunhofer considered 120 μm clinically acceptable for dental restorations cemented with polycarboxylate cement [1,5]. Even though the internal discrepancies may be well over 200–300 μm most authors conclude that the results from their *in vitro* fit studies are clinically acceptable when the mean marginal gap is below or close to 120 μm [7–11]. The tooth-crown interface is divided into different areas; marginal, chamfer, axial, and occlusal. There are several areas or distances used to assess the marginal fit of the restoration, it can be measured as the marginal gap, the vertical marginal discrepancy, the horizontal marginal discrepancy, and the absolute marginal discrepancy [12]. The internal fit can be divided into discrepancy at the chamfer or cervical area, axial discrepancy and occlusal discrepancy, or as a mean of all the measuring areas/points [13,14].

The fit of a restoration can be measured using destructive techniques, where the crown or multi-unit fixed dental prosthesis (FDP) is cemented onto dies and embedded with, for example, epoxy resin, sectioned and analyzed microscopically [15,16]. Non-destructive techniques are also used, such as; clinical examination using an explorer, direct view of the crown margin using a microscope or scanning electron microscopy (SEM) [17,18], the silicone or impression replica method [5,19], micro-computed tomography (micro-CT) [20], and optical three-dimensional (3D) scanning [21].

In order to evaluate the accuracy of a restoration, the settings for the marginal and internal spacer must be provided. Otherwise, the measurements only reflect the total deviation from the master model, however, since tooth-supported restorations seldom are manufactured with a 0 µm spacer setting, the results do not represent the accuracy. The results from the fit measurements should, therefore, be regarded as the fit, and the results minus the cement spacer setting, the accuracy.

Zirconia crowns and multi-unit FDPs are predominantly made using computer-aided design-computer aided manufacturing (CAD-CAM). A systematic review on the fit of CAD-CAM restorations of different materials found marginal gaps between 39–201 µm and internal gaps ranging from 23 to 230 µm [22].

## Aim

The aim of this study was to review the fit and assess the accuracy of tooth-supported single and multi-unit zirconia fixed dental prostheses.

## Material and method

### Search strategy

In the present study, the search was performed on 1 February 2018 in PubMed, and limited to English, Swedish, Danish and Norwegian languages published between 2 March 2013 and 1 February 2018. The searches and terms were:

(((FDP [Title/Abstract] OR fixed partial denture [Title/Abstract] OR FPD [Title/Abstract]) OR ((prosthesis [Title/Abstract] OR prostheses [Title/Abstract]) AND ((‘dental health services’ [MeSH Terms] OR (‘dental’ [All Fields] AND ‘health’ [All Fields] AND ‘services’ [All Fields]) OR ‘dental health services’ [All Fields] OR ‘dental’ [All Fields]) OR (‘dentistry’ [MeSH Terms] OR ‘dentistry’ [All Fields])))) OR (crown [Title/Abstract] OR crowns [Title/Abstract] OR bridge [Title/Abstract] OR bridges [Title/Abstract])) AND (zirconia [Title/Abstract] OR zirkonia [Title/Abstract] OR ZRO2 [Title/Abstract] OR Y-TZP [Title/Abstract] OR ‘zirconium dioxide’ [Title/Abstract] OR ‘Yttria stabilized tetragonal zirconia polycrystals’ [Title/Abstract] OR 3Y-TZP [Title/Abstract]) AND (‘2013/02/03’ [PDat]: ‘2018/02/01’ [PDat])

### Inclusion criteria

- Language (English, Swedish, Danish or Norwegian).
- Studies of tooth-supported prostheses.
- Fit assessment described.
- Measurement techniques described.
- Material (Zirconia).
- Pre-set cement spacer described.
- Chamfer or round shoulder preparations.

### Exclusion criteria

- Studies not meeting all inclusion criteria.
- Studies of implant-supported prostheses.
- Studies measuring fit after ceramic veneering.
- Studies measuring only the marginal gap.
- Studies where internal adjustments were made before a fit assessment.

### Selection of studies

**Figure 1.**
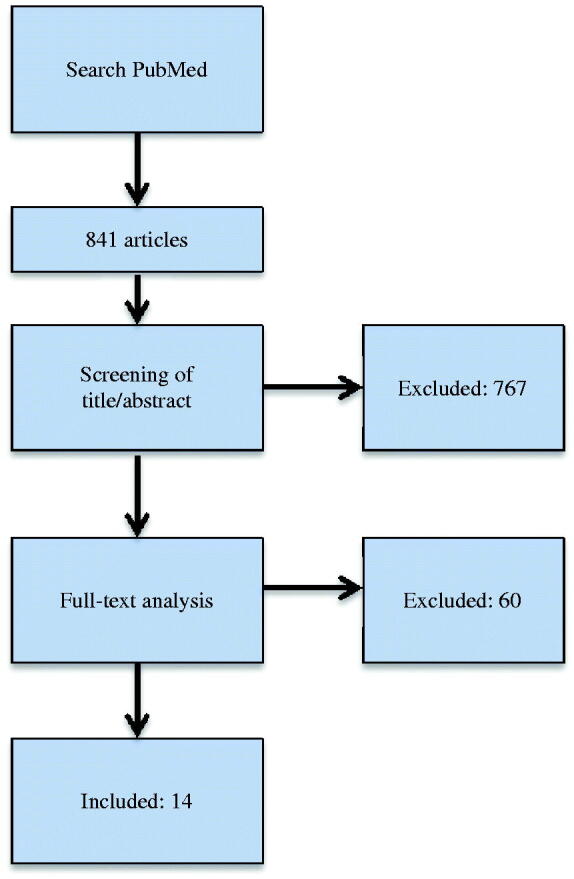
Search strategy of the systematic review. 841 articles were found and 767 were excluded after screening of title and abstract. After full-text analysis another 60 articles were excluded which left 14 articles to be included.

The titles were screened and abstracts from the studies found in the search described above, considering the inclusion criteria. After selection, the full texts of the studies were acquired. The full-text publications were screened according to the inclusion and exclusion criteria and 14 studies were included for data analysis (Figure 1 and Table 1). The data collected from the studies were; Author, Year, *in vivo*/*in vitro*, Abutment teeth, Restoration type, Restoration material, Number of specimens per group, Preparation type, Cement spacer at margin, Cement spacer internal, Impression type, Scanner, CAD software, CAM machine, Fit assessment method, Number of measuring points per abutment, Die material, Restoration manufacturing method, Marginal gap, Cervical gap, Axial gap, Occlusal gap, Internal gap and Total gap. In studies where an internal or total gap was not reported but axial and occlusal values were, the internal and total gap values were calculated by the author. In this review, Holmes et al. definition of the marginal gap were used [12]. The internal gap was the mean of all the available internal measuring points (cervical, chamfer, axial, and/or occlusal) and the total gap was the mean of all the measuring points available in the studies (marginal, cervical, chamfer, axial, occlusal).

**Table 1.** Overview of the included studies and their setting parameters and results.

	Settings						Marginal gap			Internal gap			Total gap		
Author	Impression	Abutment tooth	Units	Evaluation	Pre-set spacer	*N*	Fit	SD	Acc	Fit	SD	Acc	Fit	SD	Acc
Cetik et al. [24]	Conv	M	SC	CS	45 (1)–85	10	73	17	28	80	20	5	78	19	7
	Dig	M	SC	CS	45 (1)–85	10	63	14	18	70	15	14	69	15	16
Cunali et al. [43]	Dig*	M	SC	SR	20–70	10	78	12	58	145	36	75	128	30	58
	Dig	M	SC	SR	20–70	10	78	16	58	134	34	64	120	29	50
	Dig	M	SC	MCT	20–70	10	69	9	49	106	16	36	97	14	27
	Dig	M	SC	MCT	20–70	10	75	7	55	110	13	40	101	11	31
Dahl et al. [36]	Dig	INC	SC	3D	30 (0.5)–70	3				135	127	65	78	65	8
	Dig	INC	SC	3D	15 (0.5)–50	3				126	92	76	81	56	31
Kocaagaoglu et al. [25]	Conv	PM	SC	SR	0 (1)–30	10	86	12	86	131	17	101	116	15	86
	Dig	PM	SC	SR	0 (1)–30	10	59	20	59	127	27	97	104	25	74
	Dig	PM	SC	SR	0 (1)–30	10	48	7	48	101	13	71	83	11	53
Miura et al. [44]	Conv	M	SC	SR	0 (1)–30	5				85	29	55			
Nelson et al. [45]	Conv	PM	SC	CS	0 (1)–40	10	118	5	118	80	4	40	99	4	59
Pedroche et al. [26]	Conv	M	SC	SR	10–60	10	87	31	77	238	31	182	201	36	155
	Dig	M	SC	SR	10–60	10	59	14	49	112	33	55	95	28	53
Lee et al. [46]	Conv	INC	SC	SR	40–40	10	86	32	46	86	26	46	85	28	45
	Conv	INC-INC	4-unit	SR	40–40	10	66	24	26	124	41	84	110	37	70
	Conv	CAN-CAN	6-unit	SR	40–40	10	90	44	50	145	62	105	131	58	91
Almeida e Silva et al. [27]	Conv	PM-M	4-unit	SR	0 (0.8)–30	12	65	37	65	66	42	36	66	40	51
	Dig	PM-M	4-unit	SR	0 (0.8)–30	12	64	37	64	59	36	29	61	36	46
Dahl et al. [47]	Dig	PM-M	3-unit	3D	30 (0.5)–70	3							105	71	35
	Dig	PM-M	3-unit	3D	15 (0.5)–50	3							96	55	46
Keul et al. [9]	Conv	PM-M	4-unit	SR	30 (1.5)–60	12	141	193	111	166	138	106	160		100
	Dig	PM-M	4-unit	SR	30 (1.5)–60	12	127	67	87	154	60	94	147		87
Memarian et al. [48]	Dig*	PM-M	3-unit	CS	35–35	12	113	20	78	68	12	33	83	14	48
	Dig	PM-M	3-unit	CS	35–35	12	106	19	71	73	20	38	84	20	49
	Dig	PM-M	3-unit	CS	35–35	12	117	19	82	80	15	45	92	16	57
Su & Sun [29]	Conv	CAN-PM	3-unit	SR	40–60	10	76	18	36	134	47	74	105	36	45
	Dig	CAN-PM	3-unit	SR	40–60	10	63	16	23	110	40	50	87	28	27
Ueda et al. [28]	Conv	PM-M	4-unit	SR	30 (1.5)–60	12	87	60	57	97	51	47	95	51	49
	Dig	PM-M	4-unit	SR	30 (1.5)–60	12	63	42	33	68	33	18	67	35	22

The results for marginal, internal and total gap are mean values in μm. Pre-set spacer: setting used in CAD software for cement spacer in μm; *N*: number of test specimens; SD: standard deviation; Acc: accuracy; Conv: conventional impression; Dig: digital impression; M: molar; INC: incisive; PM: premolar; CAN: canine; SC: single crown; CS: cement section technique; SR: silicone replica technique; MCT: micro-CT; 3D: 3D scan technique. *Scanned master.

Two of the studies did not use an impression, the master model was scanned using a lab scanner and the restorations placed on the master model. The studies were included but the impression method used in those studies was grouped with the digital impression technique.

### Statistical analysis

Descriptive data are presented as numbers and frequencies. Mean values were calculated as weighted values based on the individual group mean value and the number of test specimens per group. The fit was the mean of distances reported in the studies and accuracy was the fit minus the pre-set spacer.

## Results

The fourteen studies included in the analysis for this review presented fifteen results for single crowns, seven for three-unit FDPs, seven for four-unit FDPs, and one for six-unit FDPs. Four different fit measuring techniques were used; the silicone replica technique (18 results), the cement and section technique (eight results), the 3D scan technique (Four results), and the Micro-CT technique (Two results) (Table 1). The scanners, CAM machines and zirconia materials used are described in Table 2.

**Table 2.** Overview of the intraoral scanners (IOS), laboratory scanners, CAM systems, and zirconia materials used in the included studies.

IOS system	Scanner/CAD system	CAM system	Zirconia material
3M Lava COS [27,28]	3M Lava [27,28]	3M Lava CNC 500 [27,28]	3M Lava Zirconia [28]
3Shape TRIOS [24–26,29,36,47]	3Shape D700 [26,48]	Ceramill Motion 2 [24,48]	Ceramill Zi [43,45,48]
Cerec Omnicam [25]	3Shape D800 [29]	Cercon Brain expert [44,48]	Cercon ZR [44,48]
iTero [9]	3Shape N/S [24]	Cerec MC XL [25]	Denzir [36,47]
N/S [46]	Ceramill MAP400 [43,48]	DMG Mori Ultrasonic 20 linear [26]	DD Bio ZW 3Y-TZP [36,47]
	Cercon Eye [44]	Imes iCore Coritec 250i [25]	InCoris [43]
	Cerec inEOS X5 [25]	Straumann milling [9]	Metoxit Zirkonia [26]
	Cerec inLab [43]	VHF 450 classic [45]	Straumann Zerion [9]
	Dental Wings [25,45]	Zirkonzahn M2 [48]	Upcera [29]
	Straumann Cares 2 [9]	N/S [29,36,43,46,47]	Zirkonzahn Prettau Zr [48]
	Zirkonzahn S600 Arti [48]		Zirkonzahn ICE Zirkon HT [25]
	N/S [36,46,47]		N/S [24,27,46]

N/S: not specified.

For the marginal gap of single crowns and multi-unit FDPs combined, the fit was 83 μm and the accuracy was 59 μm. The internal gap fit was 111 μm and the accuracy 61 μm. For the total gap, the fit was 101 μm, and the accuracy of the zirconia restorations was 53 μm (Table 3). Eleven of the results were for restorations made from conventional impressions and 19 results were from digital impressions (Table 4).

**Table 3.** Fit and accuracy of the zirconia restorations in μm, for marginal, internal, and total gap.

	*N*	Mean	SD	Min	Max
Marginal gap					
Fit	26	83	24	48	141
Accuracy	26	59	25	18	118
Internal gap					
Fit	29	111	39	59	238
Accuracy	29	61	36	5	182
Total gap					
Fit	30	101	30	61	201
Accuracy	30	53	30	7	155

*N*: number of test results; SD: standard deviation.

**Table 4.** Fit and accuracy of zirconia restorations in μm, for marginal, internal, and total gap according to impression technique.

	Impression		
	*N*	Mean	SD
Marginal gap			
Conv fit	11	89	23
Conv accuracy	11	64	32
Dig fit	15	79	25
Dig accuracy	15	55	20
Internal gap			
Conv fit	12	119	49
Conv accuracy	12	73	46
Dig fit	17	104	30
Dig accuracy	17	53	25
Total gap			
Conv fit	11	113	39
Conv accuracy	11	69	39
Dig fit	19	94	21
Dig accuracy	19	43	20

*N*: number of test results; SD: standard deviation; Conv: conventional impression; Dig: digital impression.

## Discussion

The purpose of this systematic review was to evaluate the fit and assess the accuracy of tooth-supported single and multi-unit zirconia fixed dental prostheses. The fit of the zirconia FDPs was within the range (max 120 μm) most researchers deem clinically acceptable [1,5,7–11]. In an earlier review on the fit of CAD-CAM restorations, published in 2014, the marginal gaps ranged from 39 to 201 µm and the internal gaps from 23 to 230 µm [22]. These results are in accordance with the findings of the present study. In this review, based on studies published between 2013 and 2018, the marginal gaps ranged from 48 to 141 µm and the internal gaps from 59 to 238 µm. An improvement in the fit results could perhaps have been hypothesized due to the developments in CAD-CAM technology. However, this could not be seen in this comparison. It may be since, in the review by Boitelle, other materials such as glass-ceramics and alloys were included which could have affected the results. The choice of restorative material has been shown to influence the marginal fit, in a study by Rödiger et al. with the same settings spacer for all materials, zirconia copings had significantly larger marginal gaps compared to titanium and cobalt-chromium copings [23]. Many of the included studies in this review aimed to compare the fit results of restorations from conventional and digital impressions [9,24–29]. When the results were compared according to the impression technique, the fit and accuracy for all three fit assessment areas were slightly smaller for the digital impression technique. This supports the results from other studies on digital impressions, where single crowns and multi-unit FDPs up to 8-units from digital impressions have shown comparable or lower fit values compared to conventional impressions [30–32].

When comparing the results from different studies one must be aware of the complexity due to the methods and parameters used [33]. In this review, all the restorations were produced using CAM-milling, however, four intraoral scanners and several laboratory scanners were used. Also, nine different milling machines were named in the studies and five studies failed to mention what machine was used. The choice of milling machine may affect the fit of restorations, Kirsch et al. compared five-, and four-axis milling machines and found higher trueness in machines with five-axes [34]. Regarding the zirconia materials used in the studies, eleven different zirconia materials were found and four were not disclosed. For the fit assessment, nine studies used the silicone replica technique, three studies used the cement and sectioning technique, two used the 3D scan technique, and one used the Micro-CT technique. These parameters would be interesting to compare using factor analysis, however, in the present review, there were too few results from each factor to conduct a meaningful analysis. Hence, the results in this review should only be regarded as descriptive. If a stricter inclusion protocol could be used and enough studies would meet the criteria, the before mentioned and other parameters such as; cementation pressure, tooth, preparation type, type of master model and material, could be compared and analyzed.

In the systematic review by Boitelle, only about 50% of the 26 included studies reported the cement spacer settings [22]. In this review only 14 studies were included of the 74 that were originally analyzed in full-text, 25 of the 60 excluded studies did not report the settings. It is important to disclose as much information as possible about the production process and fit assessment technique in the materials section since the settings and parameters may affect the results and conclusions [35]. As an example, it would be a mistake to conclude that a technique or material with a spacer setting of 40 μm and a fit result of 70 μm is more accurate than a technique or material with a spacer setting of 60 μm and a fit result of 80 μm. The first technique is 30 μm from the aimed at spacer setting and the second technique 20 μm. Therefore; the accuracy is higher in technique 20 μm from the setting. In a study by Wettstein et al., the conclusion was that metal-ceramic FDPs had significantly smaller internal gaps compared to zirconia FDPs. However, when taking the spacer into consideration, the only significant difference found was that zirconia FDPs had a significantly smaller occlusal gap [35]. Other studies report the spacer settings but fail to use them when drawing conclusions [36,37].

The majority of the included studies used the silicone replica technique for fit assessment; the advantages of this technique are that it can be used both *in vivo* and *in vitro*, and it does not require expensive equipment. The disadvantages are that it is restricted to a two-dimensional analysis of the fit and only the specific points chosen are used. Also, there is a risk of rupture of the light-body material when removing the restoration and it is important to place the restoration and section the replica correctly [6,16]. Nevertheless, the method is considered reliable, although it may overestimate the gap with two to 11% [19,38].

The 3D scan technique can provide a 3D view of the fit, which can be used for both quantitative and qualitative assessments. It can also be used to isolate and measure the fit in specific areas or sections [32,39]. However, it is unclear if studies using the 3D scan technique presents results for the absolute marginal gap or marginal gap [40]. The 3D scan technique may not be the most suitable technique for measuring the absolute marginal gap due to uncertainty in if the outermost edge of the restoration margin is captured with the scanner [41]. Measuring the marginal gap could result in a smaller gap value than represented by the absolute marginal gap, earlier studies on milled CoCr and zirconia have found absolute marginal gaps of 185–260 μm [16,32] and 94–181 μm [42].

The accuracy of zirconia FDPs was ∼60 μm for MG, IntG and TotG. The dental laboratories could perhaps use this information when designing restorations, by changing the spacer settings to improve the fit. However, these results are based on a wide range of different scanners, design software, and CAM machines, all with several parameters that can affect the fit of a restoration. The dental laboratories should do their own tests and measure the accuracy of the restorations they manufacture and adapt the spacer settings accordingly. The easiest method would be the silicone replica technique.

## Conclusions

Within the limitations of the present systematic review, the fit of zirconia single crowns and multi-unit FDPs may be regarded as clinically acceptable, and the accuracy of the manufacturing of zirconia is ∼60 μm for marginal, internal, and total gap. Also, digital impressions seem to be associated with a smaller gap value.
